# Lifestyle Interventions Improving Cannabinoid Tone During COVID-19 Lockdowns May Enhance Compliance With Preventive Regulations and Decrease Psychophysical Health Complications

**DOI:** 10.3389/fpsyt.2021.565633

**Published:** 2021-07-16

**Authors:** Viola Brugnatelli, Enrico Facco, Gastone Zanette

**Affiliations:** Department of Neuroscience, University of Padua, Padua, Italy

**Keywords:** cannabinoid, stress, HPA, resilience, lockdown, CBD, COVID-19, meditation

## Abstract

Studies investigating the psychosomatic effects of social isolation in animals have shown that one of the physiologic system that gets disrupted by this environment-affective change is the Endocannabinoid System. As the levels of endocannabinoids change in limbic areas and prefrontal cortex during stressful times, so is the subject more prone to fearful and negative thoughts and aggressive behavior. The interplay of social isolation on the hypothalamic-pituitary-adrenal axis and cannabinoid tone triggers a vicious cycle which further impairs the natural body's homeostatic neuroendocrine levels and provokes a series of risk factors for developing health complications. In this paper, we explore the psychosomatic impact of prolonged quarantine in healthy individuals, and propose management and coping strategies that may improve endocannabinoid tone, such as integration of probiotics, cannabidiol, meditation, and physical exercise interventions with the aim of supporting interpersonal, individual, and professional adherence with COVID-19 emergency public measures whilst minimizing their psycho-physical impact.

## Introduction

### The Psycho-Social Impact of Social Restriction

The current emergency public measures applied to prevent COVID-19 spread have restricted fully or partially people in lockdown cities, with half of humanity directly affected by these changes. (1) Billions of lives have been significantly altered, and a global, multilevel, and demanding stress-coping-adjustment process is ongoing.

Historically, social isolation has been used extensively as a research model for psychosis, suicide, anxiety and depression: all conditions in which the endocannabinoid system is implicated through different pathways, including the modulation of hypothalamic–pituitary–adrenal axis (HPA) function (2–4). Social isolation has been directly linked to increased incidence of suicide (5) and is considered a crucial factor contributing to suicide in humans (6–8). It induces abnormal forms of behavior including an increase in anxiety-and depressive-like activities—such as increase in immobility time, decrease in grooming activity (3), exacerbation of aggressive behavior, and impaired fear extinction (9–11)—effects which directly affect both glucocorticoid and endocannabinoid responses (12).

Although lockdowns are used for social distancing and not to “socially isolate” people, the psychopathologist Baek has introduced the concept of “socially withdrawn” to define those individuals who are isolated because of their extreme involvement in the cyber space and the lack of relationship in real life (13). One may reckon that “real life” and “cyber life” are separated by a fine line when all interpersonal and professional relationships are experienced remotely, as it is currently happening to billions of people worldwide.

Prolonged social distancing has inevitably decreased the physical expressions of affection and social bonding and altered the neuroendocrine balance of neuropeptides (e.g., oxytocin), endocannabinoids (e.g., anandamide), and corticosteroids (e.g., cortisol), key physiologic systems mutually involved not only in maintaining emotional wellbeing but also in the regulation of the immune response, making the implementation of homeostatic strategies targeting those neural pathways even more pivotal during a viral global pandemic (14).

### Lockdown: Endocannabinoid and HPA Tone Disruption

Although the endocannabinoid (eCB) system is expressed ubiquitously in the brain, the highest CB1 receptor density is distributed in key areas for the regulation of stress and emotions: prefrontal cortex (PFC), hippocampus, and amygdala (15).

Previous studies have shown that antagonism or genetic removal of CB1 induces anxiety (16, 17); on the other hand, stress, fear, and negative emotions—as many people are prone to experience since COVID-19 diffusion—may alter the expression of CB1R in the amygdala, nucleus accumbens (reward system), and PFC (18–20). As the PFC is linked to the negative modulation of aggression in animals (21, 22), social isolation may induce a PFC-specific neuronal loss, which is associated with increased levels of aggressive behavior (23).

Social isolation also impairs the hypothalamic eCB system, a brain area also involved in the onset and manifestation of aggressive and fear-related behavior in mammals (24). Studies have demonstrated that the changes in the eCB system may contribute to the display of the stress response, yielding HPA axis stimulation and increased anxiety behavior (25). Indeed, rats that are housed as isolates show an alteration of the basal HPA axis activity and impairments in glucocorticoid-mediated negative feedback (26). These neuroendocrine unbalances are paralleled in human social isolation, with studies showing that cortisol levels are higher in chronically isolated individuals (27–29).

## Psychopathology of eCB Unbalance

The endocannabinoids anandamide (AEA) and 2-arachidonoyl-glycerol (2-AG) are partial and full agonists at CB1 and CB2 receptors, respectively (30); increasing their levels *via* deletion or pharmacological inhibition of their metabolic enzyme fatty acid amide hydrolase (FAAH) was shown to reduce anxiety-like behavior (31, 32). An innate decreased anxiety-like conduct and better stress-coping behavior is associated, both in animals and in humans, with a common polymorphism in the FAAH gene and enhanced fronto-amygdala connectivity (33). Conversely, eCB levels are altered following several stressors. AEA is usually decreased, whereas 2-AG is most often increased under stressful conditions (25, 32, 34, 35). Chronic stress engenders a reduction of AEA concentration in the amygdala–hippocampal–cortico-striatal circuit, a feature commonly found in depression and posttraumatic stress disorder (PTSD) (2, 36). In these conditions, low AEA levels are associated with low cortisol levels and an upregulation of CB1 in the brain, effects which seem to be more pronounced in females than in males (37, 38). Gender differences on eCBs support the knowledge that women have a higher risk for anxiety, depression, and PTSD than men (39, 40). In addition, as reports have been showing consistently, during global health crises like COVID-19, women have been reporting higher rates of anxiety and depression than men, which relate with external stressors prevalently targeting women (increase of workload in the healthcare sector, increase of domestic burden, impoverishment, and increase in domestic violence) (41–45).

In short, endogenous cannabinoid signaling is essential for stress adaptation, while chronic stress (e.g., repeated restraint) reduces AEA levels throughout the corticolimbic stress circuit (31, 32).

These data were confirmed by studies examining the effects of social isolation on genes linked with eCB signaling, showing that this stressful condition alters several brain regions implicated in the pathophysiology of schizophrenia and PTSD, both in animals and in humans. CB1 is involved in stress regulation and result altered in several psychiatric disorders—such as anxiety, depression, bipolar disorder, PTSD, schizophrenia, attention deficit hyperactivity disorder, and eating disorders (46, 47). While prefrontal CB2 decreases after repeated stress both in males and in females, social isolation may induce upregulation of CB1R mRNA transcripts in cortical regions and downregulation in the amygdala in a gender-specific pattern due to the interaction between eCBs and sex steroids (48, 49). Females (both adolescent and adults) show higher baseline of CB1 and CB2 mRNA expression levels than males do, which is consistent among animal and human studies, as well as heighted eCB tone disruption to both acute and chronic models of stress (50–52).

Changes also occur in the regulation of eCB mRNA expression in amygdaloid regions, especially AEA, a fact associated with increased anxiety in rats reared in isolation (48, 53, 54). The COVID-19 pandemic has led to a worldwide stressful time with a dual impact: lockdown and fear of disease and death; thus, resilience and appropriate coping strategies become factors of crucial importance to reduce its impact and prevent stress switching into chronic ailments. Neurobiological observations have identified increased firing at the basolateral amygdala (BLA) and prelimbic PFC (plPFC) circuit as one of the key events in the “switch” from distress to psychiatric disorders (55). Recent findings have shown that the neuronal hyper-firing occurring in the BLA-plPFC circuit is due to the stress-induced downregulation of the eCB tone in this region; this induces an increased glutamate release, which, in turn, switches stress exposure into anxious behavior and burn-down (56).

**Figure 1:**
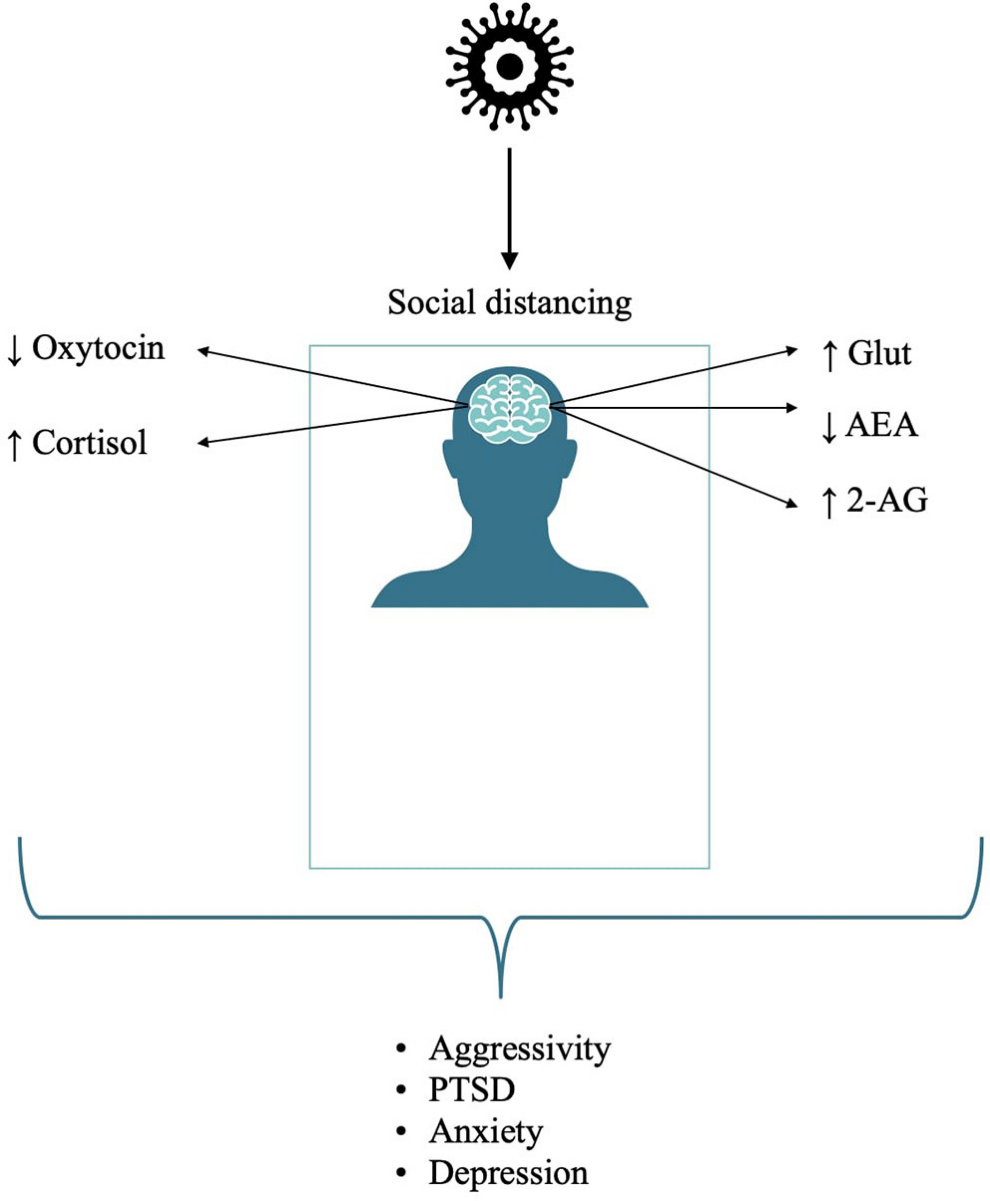
Effects of isolation on neurocircuitry and related detrimental mental health conditions.

Taken together, these data suggest the need for strategies aimed at not only decreasing stress *per se* but also helping to restore the eCB homeostasis, which is tightly bound to individual reactions in stressful environments and events (56). See Figure 1 for a summary on the effects of isolation on neurocircuitry and related mental health conditions.

### Nutraceutical Interventions

It was shown that the microbiota is tightly connected to emotional behaviors and stress-induced changes (57) and that a perturbation in the microbiota causes functional changes in the eCB tone and behavioral alterations that correlate with depressive states. Therefore, the administration of probiotics may potentially help improve both depressive-like behaviors as well as stimulate the eCB tone, increasing AEA levels (58).

Another approved dietary supplement, N-palmitoylethanolamide (PEA), a fatty acid amide belonging to the eCB system (found increased under stressful conditions), has also shown promising potential antidepressant properties alone or in combination with other classes of antidepressants (59). Preclinical studies have shown its efficacy in depression and depression associated with neuropathic pain and traumatic brain injury models, which could serve the post-COVID symptoms (60). In a translational perspective, a randomized, double-blind study in depressed patients indicated a fast-antidepressant action of PEA when associated with citalopram (59). Several foods may also help modify gut microbial metabolism and glucocorticoid productions (61). One study demonstrated that a daily consumption of 40 g of dark chocolate during a 2-week period was sufficient to reduce the stress hormone cortisol and stress-related impairments (62). Moreover, it was shown that *Theobroma cacao* contains N-acylethanolamines (N-linoleoylethanolamide and N-oleoylethanolamide) which act as FAAH inhibitors (63–65).

Other food compounds that boost the eCB tone are found in black pepper, both *via* CB2 direct agonism by one of its sesquiterpenoids, b-caryophyllene, and *via* AEA uptake inhibition by the pepper's alkaloid guineensine (66, 67). B-Caryophyllene is commonly found in spices and hence called “dietary cannabinoid” and was recently shown to also elicit antidepressant-like activity in isolated and stressed animals *via* CB2 interaction (68, 69).

Remaining in the “dark colored” food palette, *Tuber melanosporum* (black truffles) contain biosynthetic enzymes for AEA; the eCB concentration increases up to levels that have been found sufficient to activate CB1 and CB2 as the truffles' pigmentation augments (70).

Fruit and vegetable containing the flavonoid kaempferol are also recommended to balance the eCB tone as it is possible that a high dietary intake of this substance could boost serum AEA levels *via* FAAH inhibition (71). Kaempferol is commonly found in capers, *Kaempferia galanga*, saffron, arugula, blackberries, and many other edible plants (72).

Finally, although it is still debated in literature whether a dietary intake would be sufficient to produce CBR-mediated effects, curcumin, the main constituent of turmeric, was nonetheless found to decrease immobility in animal depression models (73, 74).

### Phytoceutic Interventions

During lockdown in Canada and a few US States, cannabis was declared an essential service, allowing dispensaries to continue their activity and deliver cannabis products to customers (75). The authors agree that there is a rationale for the use of cannabis during these times. Studies have shown that the depressive-like behaviors induced by social distancing are mitigated by CB receptor activation (3). Moreover, isolation induces a reduction in PFC dendritic dopamine D2 receptors which can be rescued by CB1 stimulation (76). Where cannabis was more available, an increase of its use during lockdown measures was reported, perhaps highlighting an intuitive understanding by consumers for the need to increase the eCB tone (77–81). The increased cannabis use was self-administered, yet not associated with an increase in DSM-5-CCSM total, depression, anxiety, and sleep problem scores in these Countries (78). However, as we recommend strategies that may be applied to the population at large, we would encourage the use of compounds other than tetrahydrocannabinol (THC)-containing cannabis, hence devoid of intoxicating effects. As lockdown poses an increased risk for isolation-induced aggression, the use of cannabidiol (CBD), a cannabis compound with anxiolytic, antidepressant, and antipsychotic properties, could prove useful (82). CBD has been tested in isolated animals, confirming its beneficial effects in attenuating aggressive behavior through a mechanism associated with an increase in AEA levels (*via* FAAH inhibition) and activation of 5-HT1A and CB1 receptors (83).

Another non-psychotropic plant, *Echinacea*, can interact with cannabinoid receptors, both directly *via* alkamide interaction at CB2 and indirectly *via* AEA reuptake inhibition (84, 85). Echinacea preparations, often used as a home remedy for colds, have been showing to induce an anxiolytic effect in animal models (86).

### Physical Activity

Physical activity has shown to play an important role in maintaining mental health, decreasing anxiety and alleviating depressive symptoms, and has been hence already recommended as a regular practice during the pandemic to prevent metabolic and immunological dysfunctions (87–89). Interestingly, it also modulates the eCB system balance, a fact suggesting its relevance from both a neurobiological and psychosomatic perspective (90). Indeed, increased eCB concentrations were found following a simple jog, bike ride, hike, and other moderate intensity aerobic exercises, while the differences of FAAH hydrolytic activity in active and sedentary individuals result in different AEA plasma levels (91–94).

### Hypnosis and Meditation

Meditation and hypnosis, despite their seemingly different history and conceptualization, may be regarded as two faces of the same coin, sharing several historical, procedural, and neuropsychological aspects (95, 96) as well as their link to the placebo effect (97). Placebo effect involves both endogenous opioids and the eCB system (98). As a whole, the available data on neuropsychological aspects of hypnosis and meditation highlight the relevance of a holistic approach encompassing the inseparable mind–brain–body–environment unit. A wealth of data has shown how hypnosis and meditation may enhance metacognitive control and engender intentional changes of activation/deactivation of unconscious brain areas and circuits leading to outstanding results, one for all hypnotic analgesia (99–102). Both hypnosis and meditation may help develop awareness, mindfulness, and metacognition, restructuring the patient's problem, enhance one's control over mind and body, and manage functional and psychosomatic disorders (95, 103). Therefore, one must establish a bidirectional mind–brain relationship, where brain changes yield mind ones and vice versa. As a result, both pharmacological and nutraceutical interventions may improve brain functions and help decrease symptoms, while behavioral techniques directly modulate the same brain function through cognition, a top-down mind–brain rearrangement.

A broad range of neurological and psychiatric disorders as well as deficits in self-referential processing, such as depersonalization, seem to be related to an altered balance between the default mode network, the salience network, and the central executive network (104). Their complex interplay and connection with other circuits as a dynamic whole is able to update according to demands (105), while their alterations may engender psychiatric disorders. Likewise, traumatic experiences may lead to increased activity of amygdala paralleled by a decreased capacity of anterior cingulate cortex to inhibit it (106).

On a neuropsychological standpoint, both hypnosis and meditation strongly affect the default mode network, a circuit involved in self-referential processing, and the anterior cingulate cortex, which, as mentioned above, may play a central role in dissociative identity disorders and PTSD (107). In doing so, they can help modulate the interrelationship of the abovementioned networks, which are the neuropsychological components of several psychological and psychiatric disorders and favor resilience.

## Conclusions

When life adversities and distress are concerned, like those related to COVID-19, resilience plays a key role. Resilience is a complex construct endowed with profound philosophical implications extending to Eastern culture (103); initially coined in physics and engineering, later on it has extended to both biology and psychology. Resilience is the capacity to withstand adversities by keeping the homeostasis, by recovering the initial balance following perturbation or achieve a new balance through allostasis.

At a physiological level, maintenance of homeostasis is the primary function of the eCB system. During stressful times, the eCB tone changes in limbic areas and prefrontal cortex, which makes subjects more prone to a fearful state, negative thoughts, and aggressive behavior.

**Figure 2:**
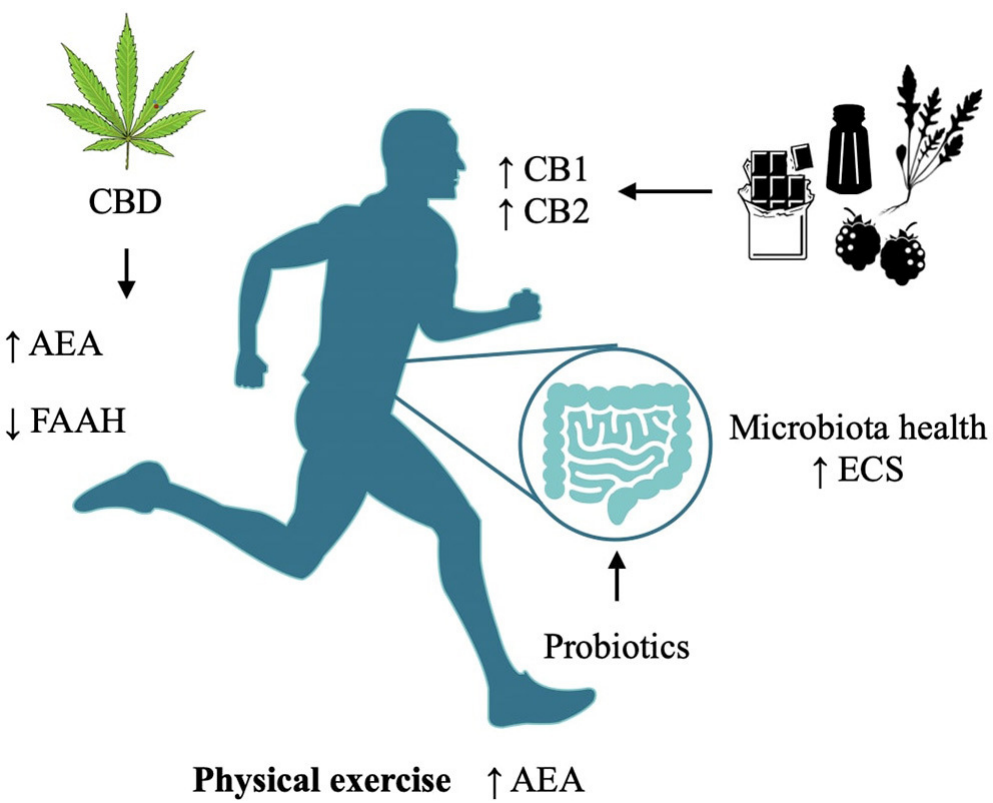
Modulation of the Endocannabinoid System via different methodologies and their targets.

On a psychological standpoint, resilience is the capacity to withstand or quickly recover from difficult conditions, such as those related to family and social relationships, financial stressors, and workplace and health problems; it is a dynamic process of both biological and psychological adaptation, including emotional and intellectual aspects and their management in the sociocultural and environmental interaction that allows a better adaptation and coping by cognitive improvement and self-transformation, features reflected in the brain through their interplay with neurotransmitter regulation. This is the rationale for presenting different lifestyle interventions and holistic approaches that integrate phytotherapy and nutraceutical agents together with behavioral techniques—like hypnosis and meditation—which are presented with the aim of spreading preventive awareness on maintaining a healthy eCB system, especially during stressful situations like COVID-19. See Figure 2 for a summary of the interventions. A detailed analysis of each of the therapies is far beyond the limit of this article; instead, we discuss first the knowledge drawn from the neurocorrelates of psychological and psychiatric disorders, and then we outline a few essential aspects of their implication in relation to the current state of emergency. We aim to support governments worldwide in using evidence-based suggestions and indications to manage the socioeconomic problems generated by poor mental health.

## Data Availability Statement

The original contributions presented in the study are included in the article/supplementary material, further inquiries can be directed to the corresponding author/s.

## Author Contributions

VB conceived and designed the study, and wrote the first draft of the manuscript. VB and EF wrote sections of the manuscript. All authors contributed to manuscript revision, read, and approved the submitted version.

## Conflict of Interest

The authors declare that the research was conducted in the absence of any commercial or financial relationships that could be construed as a potential conflict of interest.
